# Analysis of the Plastid Genome Sequence During Maize Seedling Development

**DOI:** 10.3389/fgene.2022.870115

**Published:** 2022-04-26

**Authors:** Diwaker Tripathi, Delene J. Oldenburg, Arnold J. Bendich

**Affiliations:** Department of Biology, University of Washington, Seattle, WA, United States

**Keywords:** chloroplast, chromosome, DNA damage, ptDNA, replication-transcription collisions, RNA editing, sequence variant

## Abstract

Shoot development in maize progresses from small, non-pigmented meristematic cells to expanded cells in the green leaf. During this transition, large plastid DNA (ptDNA) molecules in proplastids become fragmented in the photosynthetically-active chloroplasts. The genome sequences were determined for ptDNA obtained from *Zea mays* B73 plastids isolated from four tissues: base of the stalk (the meristem region); fully-developed first green leaf; first three leaves from light-grown seedlings; and first three leaves from dark-grown (etiolated) seedlings. These genome sequences were then compared to the *Z. mays* B73 plastid reference genome sequence that was previously obtained from green leaves. The assembled plastid genome was identical among these four tissues to the reference genome. Furthermore, there was no difference among these tissues in the sequence at and around the previously documented 27 RNA editing sites. There were, however, more sequence variants (insertions/deletions and single-nucleotide polymorphisms) for leaves grown in the dark than in the light. These variants were tightly clustered into two areas within the inverted repeat regions of the plastid genome. We propose a model for how these variant clusters could be generated by replication-transcription conflict.

## Introduction

The 140-kb plastid genome in *Zea mays* (maize) is rather typical for most plants. The genome contains a prominent inverted sequence (the “IR”) region that separates the large and small single-copy regions (LSC and SSC, respectively), and the genomic map is commonly represented in circular form because restriction fragment mapping and subsequent genome sequence assembly both predicted a circular molecule (Larrinua et al., 1983; Sugiura, 1992; Maier et al., 1995; Bosacchi et al., 2015). Analysis of DNA molecules prepared using in-gel preparation methods, however, showed that most of the plastid DNA (ptDNA) mass from non-green maize meristematic tissues was present in linear and branched-linear molecules larger than the size of the genome, and genome-sized circular molecules represented from 0 to 6% of ptDNA mass for several stages of seedling development (Oldenburg and Bendich, 2004a). When green leaves were examined, most isolated chloroplasts contained little detectable DNA, and only highly fragmented ptDNA molecules were obtained (reviewed in Oldenburg and Bendich, 2015). In contrast, linear genomic oligomers and branched forms, as well as a few circular molecules, were found for ptDNA from the leaves of seedlings grown in the continuous dark (Oldenburg et al., 2006). These results were interpreted to mean that the ptDNA was damaged by reactive oxygen species (ROS) produced as a byproduct of photosynthesis and that the ptDNA fragments represented unrepaired degradation products of the plastid genome. More recently, we reported on the end structures of linear ptDNA molecules (Oldenburg and Bendich, 2016). We also found that ROS levels increased, and DNA damage-defense measures decreased during plastid development from basal meristem to green leaf (Tripathi et al., 2020; Tripathi et al., 2022).

Another feature of ptDNA in maize and other plants is RNA editing. In the maize plastid genome, there are 27 sites where the information in DNA must be altered at the level of the RNA transcript (by changing a cytosine (C) to an uracil (U)) in order to produce a functional transcript (Maier et al., 1995; Peeters and Hanson, 2002). However, differences in the frequency of RNA editing at specific sites among tissue types, as well as reduced editing levels in non-photosynthetic tissues were reported for maize plastids (Peeters and Hanson, 2002). Since the reference genome sequence for maize ptDNA was obtained from green leaves (Maier et al., 1995; Bosacchi et al., 2015), it is possible that the sequence of the plastid genome might differ between proplastids in the non-green cells of the basal meristem and chloroplasts in green cells of the leaf. In particular, is there any change in ptDNA sequence near sites of RNA editing?

In this study, we asked whether changes in the plastid genome sequence could arise during maize development from proplastids in the basal meristem to mature chloroplasts in the leaf blade. We also compared ptDNA sequences from light-grown and dark-grown leaves. Despite the conversion of long ptDNA molecules in the basal meristem to the highly-fragmented and damaged molecules in green leaves, the plastid genome sequence we obtained was the same for proplastids, etioplasts, and chloroplasts and the same as the previously-published reference genome for chloroplasts. We did, however, find two genomic regions with variant clusters of insertions/deletions (Indels) and single-nucleotide polymorphisms (SNPs) only in dark-grown leaves. These clusters may arise from the collision between the apparatuses conducting replication and transcription of ptDNA. The data indicate that although the DNA sequences of the two IRs are identical, the two IRs (known as IRa and IRb) are not identical in function.

## Materials and Methods

### Plant Materials

Maize [(*Zea mays* L.), inbred line B73] seeds were initially obtained from the Maize Genetics Cooperation Stock Center Catalog of Stocks, Agricultural Research Service (https://data.nal.usda.gov/dataset/maize-genetics-cooperation-stock-center-catalogstocks), and plants were propagated annually to provide seed for subsequent experiments. Seeds were soaked in water overnight and sown in sunshine soil Mix #4 and vermiculite (1:1 ratio). The seedlings were grown for 12 days with a 16 h light/8 h dark photoperiod (light-grown) or in the continuous dark for 12 days (dark-grown). The light intensity was ∼500 μmol s^−1^ m^−2^ PPFD (Photosynthetic Photon Flux Density). Seedlings were washed with 0.5% sarkosyl for ∼3 min and then rinsed with distilled water. For each assay, tissue was harvested from ∼100 plants. Stalk lower (base of stalk 5 mm above the node) and leaf blades (L1 or L1 + L2 + L3) were used for plastid isolation. Stalk tissue was composed of several concentric rings of leaves, the outermost being the first leaf sheath. L1 was the fully expanded blade, whereas L2 and L3 were still developing. The coleoptile was removed before plastid isolation.

### Isolation of Plastid DNA

Plastid DNAs were extracted using cetyltrimethylammonium bromide (CTAB) as described by Rogers and Bendich (1985) with minor modifications (Tripathi et al., 2020). An equal volume of 2x CTAB buffer [2% CTAB (w/v), 100 mM Tris/HCl (pH 8.0), 20 mM EDTA, 1.4 M NaCl, 1% polyvinylpyrrolidone (M 40000; w/v); preheated to 65°C] and proteinase K (20 μg/ml) were added to the suspended plastids and the suspension was incubated at 65°C for 1 h. Then 0.1 M phenylmethylsulfonyl fluoride was added, followed by incubation at room temperature for 1 h. Then RNase A was added to 100 μg/ml, and the samples were kept at 60°C for 15 min. Next, potassium acetate was added to 400 mM, and the mixtures were kept on ice for 15 min before centrifugation at 12,000 × g for 10 min at 4°C. Equal volumes of chloroform: isoamyl alcohol (24:1) were added, the tubes were shaken, and then centrifuged at 12,000 × g for 1 min. After isopropanol precipitation, the DNA pellet was suspended in 10 mM Tris (pH 8), 1 mM EDTA (TE), and precipitated with two volumes of 100% ethanol overnight at −20°C before pelleting. DNA pellets were washed three times with 70% ethanol, dried, and then resuspended in TE. Quantitation was performed using the Quant-IT DNA quantitation kit (Thermo Fisher Scientific).

### Next-Generation Sequencing

Plastid DNAs were sequenced to assess changes and variants in DNA samples. 1–10 ng of plastid DNA from leaf and stalk tissues was used for library constructions using the Nextera XT DNA Library Preparation Kit (Illumina Inc.) at Northwest genomics center, University of Washington, Seattle. Briefly, DNAs were fragmented in 200–300 bp length by Covaris Acoustic System. The DNA fragments were then processed by end-repairing, A-tailing and adaptor ligation, a 4-cycle pre-capture PCR amplification, and targeted sequences capture. Captured DNA fragments were amplified by 15 cycles post-capture PCR. The final products were sequenced with 150-bp paired-end reads on the Illumina HiSeq X platform according to the standard manual. The raw data produced on HiSeq X were filtered and aligned against the *Z. mays* subsp. mays cultivar B73 chloroplast, (NCBI # KF241981.1) using the Burrows-Wheeler Aligner (BWA, Illumina Inc.) as described by Illumina. For read-depth analysis, we used the Samtools analysis as described on http://www.htslib.org/and in the Samtools Depth manual (http://www.htslib.org/doc/samtools-depth.html).

### Sanger Sequencing

Plastid DNAs were isolated from maize L1 (leaf 1) and stalk lower tissues from the 10-day-old seedlings grown in light as described earlier. A ∼3,000 base pair region including the *ndhB* gene was amplified by PCR using Platinum^®^ *Taq* DNA Polymerase High Fidelity (Thermo Fisher Scientific) and primers ndhB-F1 (5′-AAG​CAT​CCC​AAA​AGC​GTC​C-3′) and ndhB-R10 (5′-CAA​AAG​CAG​GTC​TGA​TTA​CAC​C-3′) (Supplementary Figure S1). The purified PCR-DNA fragments were then sent for Sanger sequencing (GeneWiz). Two sequencing primers, forward ndhB-F2 (5′-CTC​ATA​GAA​TGG​CAG​AGG​C-3′) and reverse ndhB-R8.

(5′-GGT​AAA​AGT​TCT​GTC​TTG​GTC​G-3′) were used. The subsequent sequences were aligned to the *ndhB* reference genome sequence using MacVector software. For each tissue, three biological replicates of ptDNA from each tissue were sequenced.

### IGV Analysis

Plastid genome sequence comparisons were conducted as directed in the instructions provided by Integrative Genome Browser (IGV) (https://igv.org) (Robinson et al., 2011). Briefly, the reference genome of the *Z. mays* chloroplast (NCBI # KF241981.1) with associated data files were loaded on the IGV genome browser. The reference genome sequence was shown as colored letters (A, T, C, G). Finally, the sequencing reads files (.bam) of each sample including their index files were loaded on the browser for alignment and comparison of reads with the reference genome.

### Variant Analysis

We performed variant analysis using the Illumina DRAGEN v3.8 platform as described by the manufacturer (Illumina Inc.), including the DRAGEN-GATK procedures for preprocessing the raw reads and germline variant discovery. The procedure steps are outlined in Supplementary Figure S2. Briefly, raw sequences from all samples were paired-end mapped to the reference genome of the *Z. mays* chloroplast (NCBI # KF241981.1), and genome-wide SNP and INDEL variants were called using the DRAGEN variant calling v3.8 with a filter passing variants with a Phred-scaled quality score over 30. Variant caller mode was set for the end-to-end operation for read trimming and marking duplicate reads. The final variants were shown in Variant Call Format (VCF) files (Supplementary material S2). For each tissue type and the two biological replicates, unique and/or common variants were grouped into discrete Variant Sets as described in Results and given in Supplementary material S1. A few variants did not pass the DRAGEN QUAL filter, and these were not included in the data presented here.

## Results

For our study, we used the maize inbred line B73 plastid genome as the reference genome (accession KF241981.1). B73 is among the so-called stiff-stalk germplasm group (Romay et al., 2013) and is the inbred line that was used for sequencing of the nuclear genome (Schnable et al., 2009). The size of the B73 plastome is 140,447 bp, consisting of identical IRa and IRb regions of 22,748 bp separating the LSC (82,355 bp) and 12,536 bp SSC regions (Bosacchi et al., 2015). The generally accepted convention for the plastid genome sequence numbering is to begin with nucleotide 1 (nt 1) in the LSC (just upstream of the *psbA* gene) and ending in the IRa (for genomes without IRs, the end is in the equivalent of the SSC). These, however, are not the actual ends/termini for a genome-sized monomer molecule. Restriction digestion and pulsed-field gel electrophoresis (PFGE) revealed that the DNA in the plastids of *Z. mays*, *Nicotiana tabacum*, and *Medicago truncatula* was composed of a collection of linear isomers with different ends (Oldenburg and Bendich, 2004b; Scharff and Koop, 2006; Shaver et al., 2008). For maize, the precise ends, End1 and End2, were identified by cloning and sequencing and shown to reside in the IRs (Oldenburg and Bendich, 2016). Diagrams are shown in Supplementary Figure S3 for the reference genome with nt1 in the LSC and for four isomers with actual ends. A diagram of the isomer designated as IRa-End1-Iso2 is shown in Figure 2A and subsequent figures below, although the B73 reference nt numbering (scale in Figure 2) is used throughout to indicate location/position of specific sites of interest.

PtDNA was prepared from plastids that were isolated from four different maize seedling tissues (see Materials and Methods). Leaves_light and Leaves_dark were from the first three leaves (L1+L2+L3) that were grown under 16h/8h light/dark and continuous dark conditions, respectively. Stalk_light was from the basal region of the stalk, L1_light was the fully-expanded first leaf, and both tissues were excised from seedlings grown under a 16/8 h light/dark regime. Next-Generation Sequencing (NGS) using the Illumina system was performed with ptDNA from two biological replicates for each of the four tissue types (see Materials and Methods).

### Alignment of Reads and Read Depth Analysis

**FIGURE 1:**
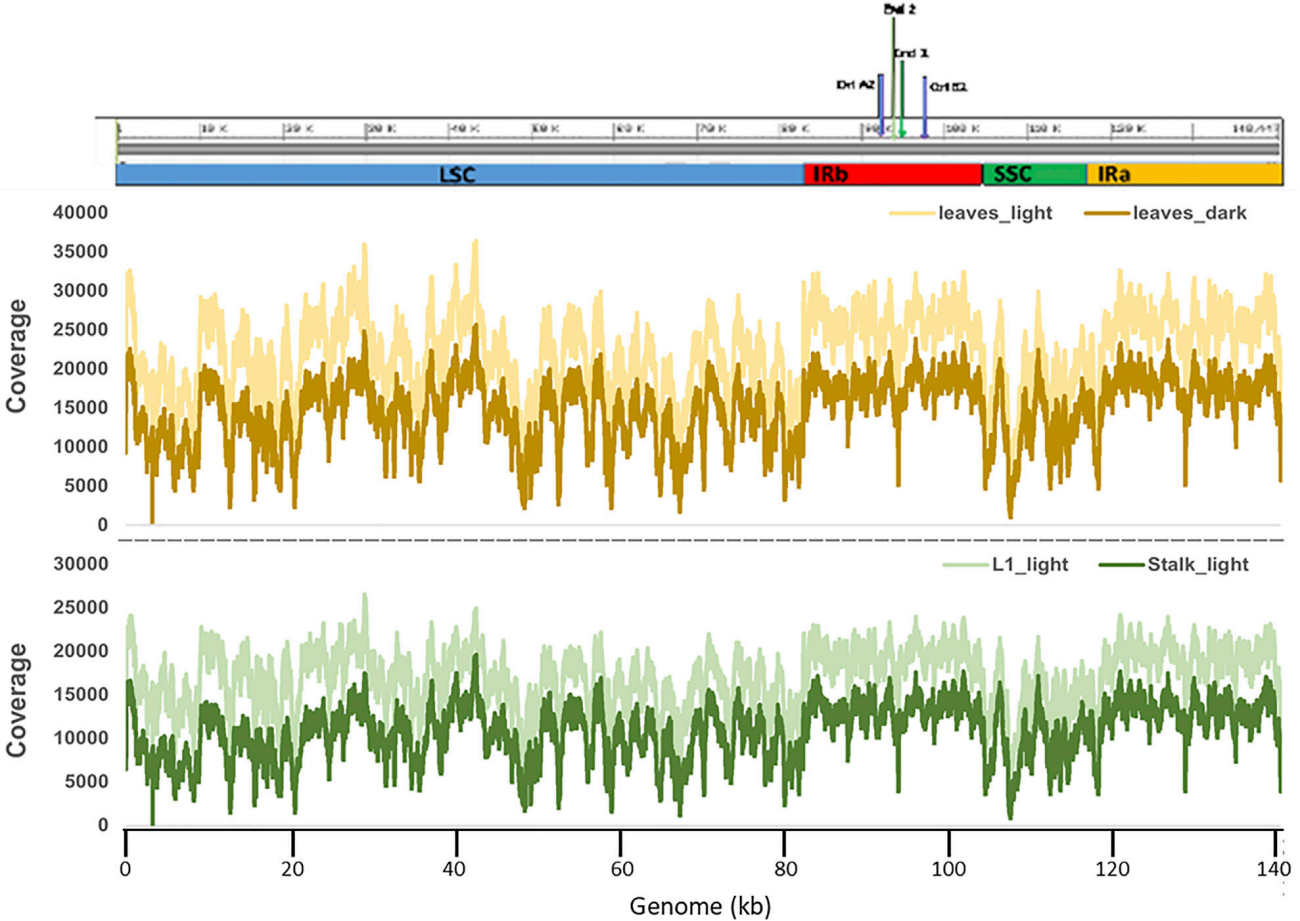
Read depth of coverage for Illumina sequencing of plastid DNA from four maize tissues. Graphs show the read depth (coverage) trend of all four samples along the entire length of the plastid genome. The upper panel shows a schematic representation of the *Z. mays* B73 plastid reference genome (NCBI # KF241981.1) with the four main regions indicated: LSC in blue; IRb in red; SSC in green; and IRa in yellow. The middle panel shows the comparison of read depths of the Leaves_light and Leaves_dark ptDNA samples. The bottom panel shows the comparison of read depths of L1_light and Stalk_light samples. The bottom *x*-axis gives the nucleotide (nt) numbering following the convention of nt1 at the beginning of the LSC.

The sequence reads were aligned against the chloroplast reference genome using the Illumina BWA analysis. Complete alignment with the reference was found with the ptDNA from all four tissue types, and a summary of results is shown in Supplementary Table S1. The read depth at all bases was also calculated in order to assess variation in read depth at any particular base or string of bases. There was some difference in the average total depth of coverage among the four samples, but as shown in Figure 1, overall the read depths followed the same patterns at all sequence positions for all four ptDNA samples: Leaves_light, Leaves_dark, L1_light, and Stalk_light. These results show that change in read depth along the plastid genome is not noticeably different during seedling development or for the light/dark growth conditions.

### Nucleotide Sequence Comparison Among Samples at RNA Editing Sites

**FIGURE 2:**
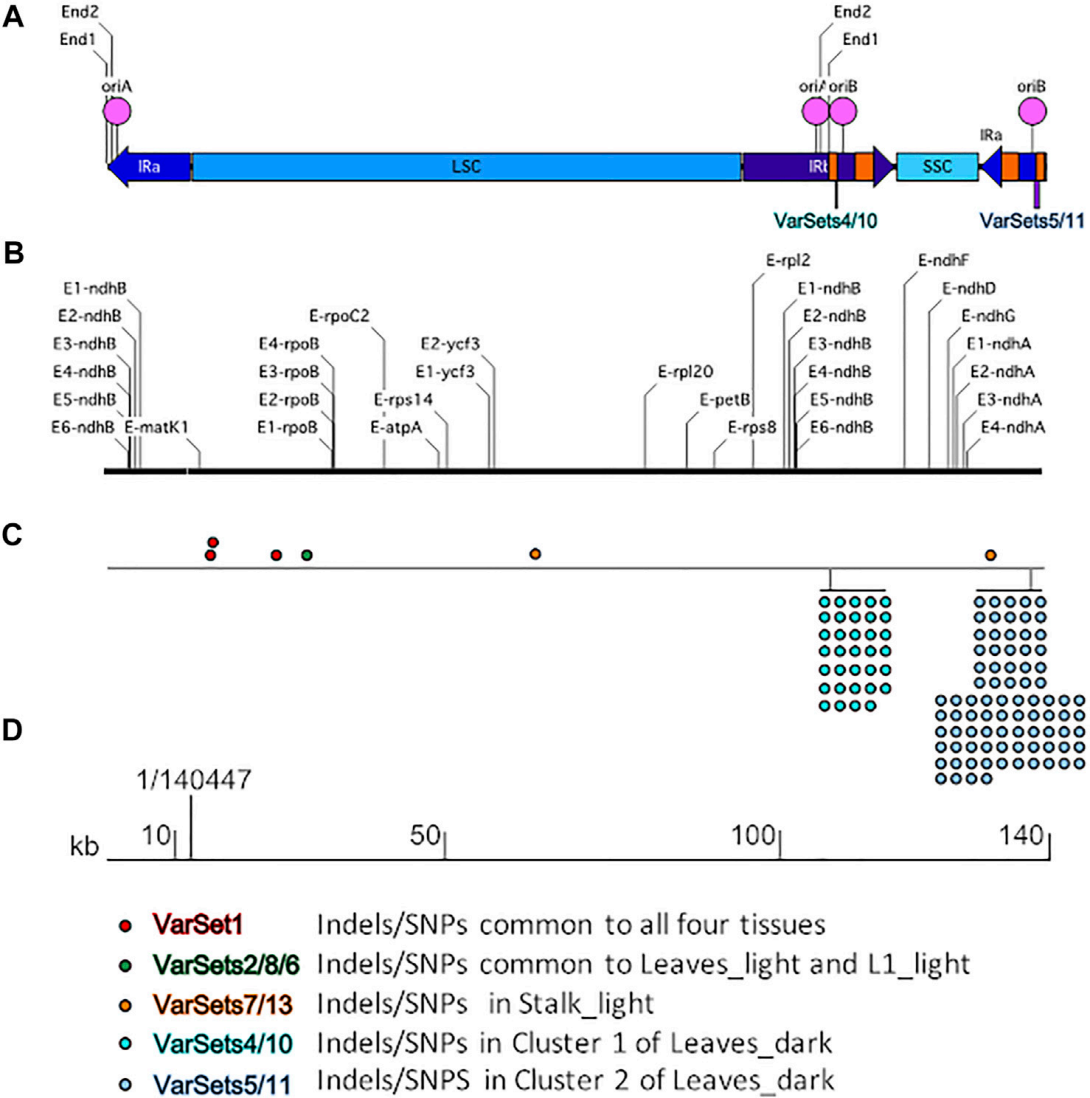
Schematic to show the RNA editing sites and variants in the maize plastid genome. **(A)** The linear isomer End1-IRa-Iso2 of the plastid genome where the left and right ends are located in the IRa region. The primary regions LSC, SSC, IRb, and IRa are indicated by blue bars and labeled accordingly, the orange bars indicate the location of the 23S (wide bar) and 16S (narrow bar) rRNA genes, the pink circles indicate the location of the origins of replication oriA and oriB, the lines indicate the location of Ends one and two in both IRa and IRb, and locations of the two variant clusters are indicated by purple bars. **(B)** The RNA editing sites. See Supplementary Figure S4 for list of sites and location on the plastid genome. **(C)** Variant sets on the genome. Color coding of the dots and corresponding tissue where the variants were found is shown at the bottom. **(D)** Scale in kb and the 1/140,447 indicates the position of the reference genome beginning/end nucleotides. The variant sets shown here are predicted by Illumina DRAGEN 3.8 variant caller software. Only variants that passed the DRAGEN QUAL filter were used in the analysis.

Although specific RNA editing sites, such as within the *ndhB* gene (Maier et al., 1992), had been reported for the maize plastid genome, a full list of putative and 25 confirmed sites was only compiled after the first complete maize plastid sequence was published by Maier et al. (1995) (accession NC_001666). A total of 27 RNA editing sites are now recognized (Peeters and Hanson, 2002). Although some differences were noted between this first plastid sequence (Maier et al., 1995) and the more recent B73 reference genome (Bosacchi et al., 2015), all 27 sites are present in both genome sequences. In general, the locations of the RNA editing sites are scattered throughout the plastid genome and present in all three major regions, the LSC, SSC, and IRs. In some cases, multiple sites are found in specific genes (Figure 2B and Supplementary Figure S4).

**FIGURE 3:**
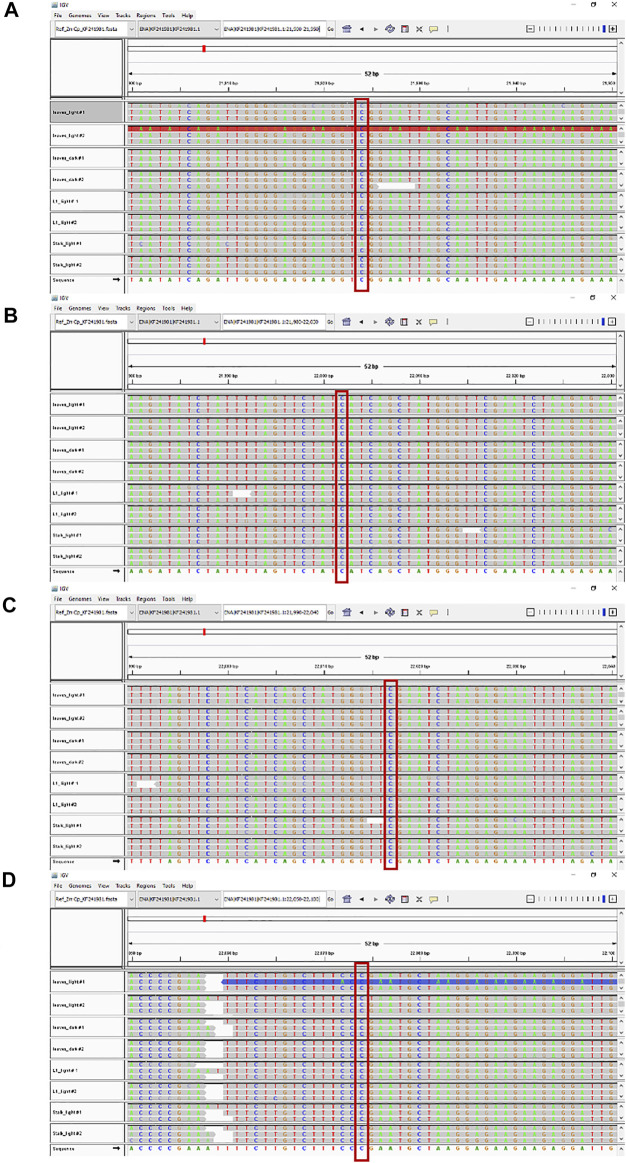
RNA editing sites within the *rpoB* gene. Maize ptDNA sequences from four tissues were aligned against the *rpoB* gene of the B73 plastid reference genome (NCBI # KF241981.1) using the Integrative genome browser (IGV). Comparison of sequences around four RNA editing sites are shown and located at nts **(A)** 21924, **(B)** 22002, **(C)** 22017, and **(D)** 22074, respectively. The conserved RNA editing sites are shown in red boxes.

IGV tools were used to compare the nucleotide sequences for the four tissue samples with the reference *Z. mays* B73 plastid genome at all 27 known RNA editing sites. An example is shown in Figure 3. For all four samples, the read alignments around the *rpoB* gene location have the same nucleotide (C) at these RNA editing positions. The same result (C at RNA editing positions) was found at all 27 RNA editing sites across the plastid genome.

**FIGURE 4:**
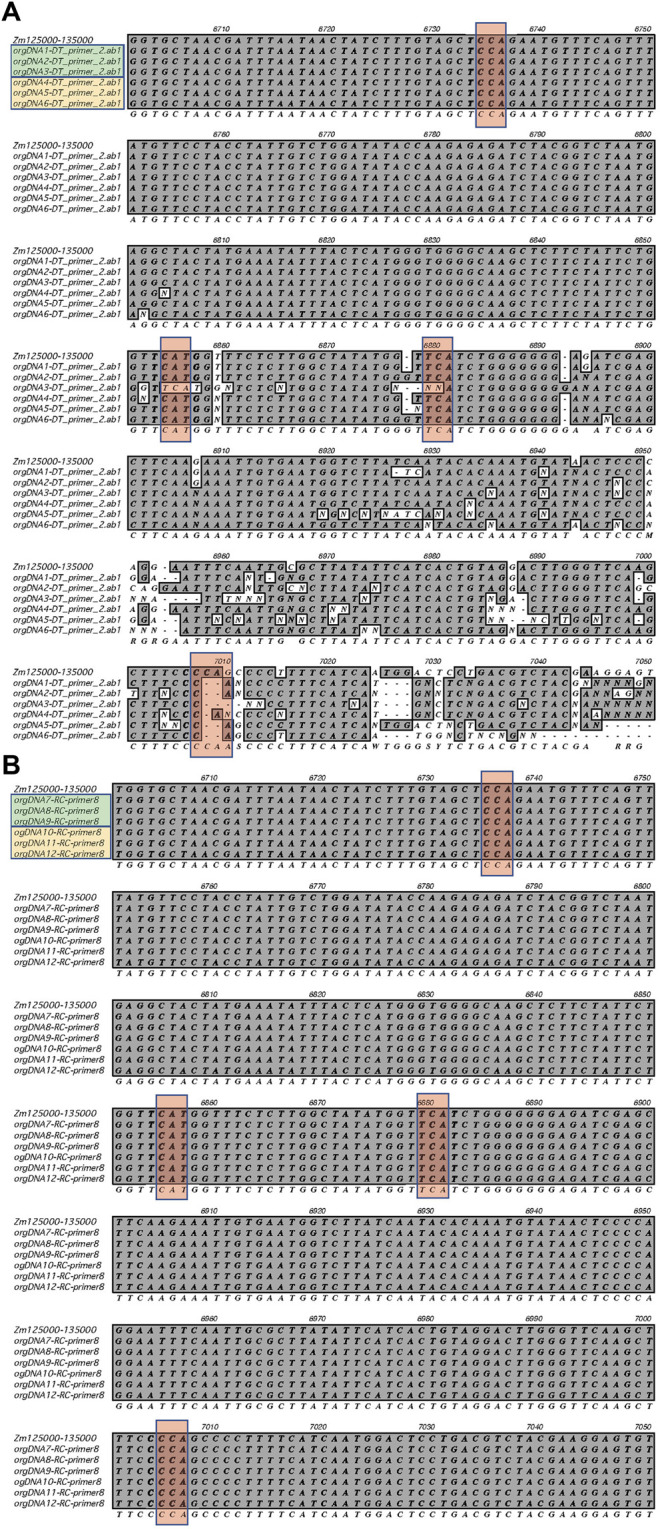
RNA editing sites within the *ndhB* gene. Sanger sequencing was performed using ptDNA obtained from L1_light and Stalk_light tissues. **(A)**The sequences obtained using forward primer ndhB-F2 are shown. **(B)** The sequences obtained using the reverse primer ndhB-R8 are shown (reverse compliment was used for alignment). Row one shows the reference sequence for the *ndhB* in IRa (+strand); Rows two to four show sequences from three samples of L1_light (green box); Rows five to seven show sequences from three samples of Stalk_light (yellow box); Row eight shows the consensus sequence. The conserved RNA editing sites are shown in red boxes.

To further examine the RNA editing sites, we performed Sanger sequencing of ptDNA from L1_light and Stalk_light tissues. The *ndhB* gene contains six editing sites, so we focused on this region (Supplementary Figures S1,S4). First, a ∼3,000 base pair segment of the plastid genome that spans the *ndhB* gene was amplified by PCR using proof-reading Taq polymerase. Then both forward and reverse sequencing primers were used to cover the first exon of the *ndhB* gene, which contains four editing sites (see Supplementary Figure S4). The nucleotide sequence alignment at RNA editing sites within the *ndhB* gene (Figure 4) shows that there are no differences between the reference genome and the ptDNA from the L1_light and Stalk_light samples. Thus, both the NGS and Sanger sequencing data show that there are no differences at RNA editing sites during seedling development for the stalk and leaf or for growth in light and dark conditions.

### Variant Analysis

Several variant-calling programs have been developed that are based on sequence-read alignment. Most include three steps: 1. Preprocessing of sequence reads; 2. Alignment (mapping) of reads to a reference sequence; and 3. Calling/identification of sequence variants based on alignments. The precision and sensitivity of the final variant set depend on the particular program and algorithm (Schilbert et al., 2020). Variant callers such as SAMtools (Li et al., 2009), Genomic Analysis ToolKit (GATK) (McKenna et al., 2010), FreeBayes (Garrison and Marth, 2012), VarDict (Koboldt et al., 2009), and VarScan (Pucker and Schilbert, 2019) use different approaches to call variants (reviewed in Schilbert et al., 2020). The recent introduction of DRAGEN (Dynamic Read Analysis for GENomics) has improved the speed and accuracy of genomic data processing using the GATK-BWA pipeline (Illumina Inc.). Schilbert et al. (2020) evaluated the performance of 50 variant calling procedures using six *Arabidopsis thaliana* data sets and concluded that GATK was the procedure of choice. GATK was also shown to be useful for variant analysis with crop plants, including rice, soybean, and tomato (Wu et al., 2019).

We performed variant analysis using Illumina DRAGEN version 3.8 software to investigate possible ptDNA sequence differences among the four tissues. The output VCF files generated all the variants present in each sample (Supplementary material S2). For our analysis, we used only those variants that passed the DRAGEN QUAL filter. Only a few variants (4 Indels/SNPs) were found in three of the tissue samples, Leaves_light, L1_light, and Stalk_light (Table 1). In contrast, many more variants (77–112 Indels/SNPs) were identified in the ptDNA from Leaves_dark tissue. In order to determine whether there were any patterns, such as Indels/SNPs common among different samples or grouping of Indels/SNPs in specific regions of the plastid genome, the variants were sorted into Variant Sets (VarSet) (Figure 2C). The Indels/SNPs in each VarSet, as well as the specific location (nucleotide) on the genome and change from the reference sequence, are given in Supplementary Tables S2,S3. There were three Indels that were common to all four tissues, and these were placed into VarSet1. This result suggests that over time there has been a slight divergence in the plastid genome of the B73 plants we grew compared with those that were used to generate the B73 plastid reference genome. Indels/SNPs that were unique to a specific tissue were then classified into individual VarSets. There was one variant found in both VarSets2/8 of Leaves_light and VarSet6 of L1_light, and for Stalk-light, there were two in VarSets7/13. Most Indels/SNPs in the VarSets defined above were located in the LSC with one in IRa (Figure 2C). For the Leaves_dark, two major clusters of variants located in the IR regions were identified: Cluster one comprised of VarSets4/10 and Cluster two comprised of VarSets5/11. Cluster one contains 34 variants that span a 200-nucleotide region in IRb, whereas Cluster two contains 84 variants that span a 549-nucleotide region in IRa. For the two biological replicates of the Leaves_dark ptDNA, there were some Indels/SNPs unique to each replicate sample, but also a large number that was common to both replicates in both Cluster one and Cluster 2, as shown in Supplementary Table S3. Note that Indels/SNPs common to both replicates were counted as single variants and indicated by single dots on the diagram in Figure 2C.

**TABLE 1:** DRAGEN variant analysis—the number of Indels/SNPs in each ptDNA sample.

	Light #1	Dark #1	Leaf #1	Stalk #1
SNPs	0	66	0	0
Indels	4	46	4	4
Total variants	4	112	4	4
	**Light #2**	**Dark #2**	**Leaf #2**	**Stalk #2**
SNPs	0	40	0	0
Indels	4	37	3	4
Total variants	4	77	3	4

In summary, there were no differences found in the assembled plastid genome sequence during development from proplastid to mature chloroplast. For both L1_light and Stalk_ light tissues, the ptDNA sequence aligned fully with the B73 reference genome and all of the 27 RNA editing sites were detected. Only minor differences for Indel/SNP variants were noted. Alignment to the reference genome and identification of the RNA editing sites were also found for the Leaves_dark ptDNA; however, there were 20–30 times more variants found for dark-grown leaf tissue than light-grown leaf tissue. Moreover, the variants were tightly grouped into two distinct areas within the IRs.

## Discussion

We present sequencing data for DNA obtained from plastids isolated from different tissues during maize seedling development. Three aspects of the data will be considered.

Although almost 4,000 plastid genome sequences are now available (CpGDB, chloroplast genome database) (Singh et al., 2020), it has evidently been assumed that the plastome sequence is invariant among tissues and that the plastome in germline cells does not differ from the plastome in somatic cells. Here, we report that the assembled plastid genome sequence for the B73 strain of maize is exactly the same among each of the four tissues analyzed and is exactly the same as that in the reference plastid genome produced from light-grown B73 maize leaves. Nonetheless, the ptDNA molecules obtained from chloroplasts isolated from green leaves are highly degraded with no detectable molecules even approaching the size of the genome (140,447 bp for B73 maize), whereas molecules at or larger than the size of the genome account for most of the ptDNA mass in basal meristem cells (Oldenburg and Bendich, 2015). If we assume that the contribution to full plastid function from plastid chromosomal DNA molecules cannot be served by highly-degraded ptDNA molecules that carry unrepaired damage (Kumar et al., 2014; Tripathi et al., 2020; Tripathi et al., 2022), then we need to consider how the sequence assembly process produces the same genome from degraded (and probably functionally impaired) ptDNA molecules and undegraded ptDNA molecules.

The second aspect of the data concerns RNA editing. Editing converts non-functional sequence information in DNA to a functional sequence at the level of the RNA transcript.

For the third aspect, our data confirm previous conclusions that the two IR regions in the maize plastome are identical in sequence throughout their length of 22,748 bp. However, analysis of sequence variants leads to a model in which the two sequences (IRa and IRb) are not equivalent with respect to their transcription and replication.

### Genome Sequencing From Intact and Degraded Maize ptDNA Molecules

The Illumina next-generation sequencing technology was used to sequence the DNA from maize B73 plastids isolated from light-grown basal stalk and leaves and from dark-grown leaves. The BWA alignment summary (Supplementary Table S1) shows that all four samples were aligned (with approximately 99% of paired reads) with the reference plastid genome assembly. The finding of the “wild type” B73 reference sequence for both intact and fragmented ptDNA may be explained as follows. First, both the Sanger and NGS methods require breaking the DNA molecules *in vitro* to small sizes suitable for sequencing. The pieces with overlapping sequences are then assembled to generate the entire plastid genome sequence. Thus, *in vitro* fragmentation would be done for the linear genomic oligomers and multi-genomic branched forms of ptDNA from the basal stalk and dark-grown leaves prior to sequencing. In contrast, the ptDNA from light-grown leaves has already been fragmented *in vivo* to less-than-genome sized pieces, although the production of smaller pieces by *in vitro* processes may be necessary for NGS. Consequently, both intact and *in vivo*-fragmented ptDNA would yield sequence that would assemble and align to match the reference genome.

Second, the types of damage to the ptDNA in green leaves may include double-strand breaks, sections with single-strand DNA, degradation by endonuclease, and bulky lesions (Kumar et al., 2014). Some of these types of damage may impede the progression of *Taq* DNA polymerase, so that only impediment-free fragments would contribute to genome assembly and identical alignment with the reference genome. Thus, the assembled plastid genome is identical for both intact and highly fragmented/damaged ptDNA, even though only the intact form in proplastids and etioplasts contributes to full plastid function.

### RNA Editing

In general, angiosperms contain 30–50 RNA editing sites in their plastid genomes (Oldenkott et al., 2014). Twenty-seven RNA editing sites have been identified in maize plastids (Maier et al., 1995; Peeters and Hanson, 2002; Bosacchi et al., 2015). After aligning the reads with the plastid reference genome, we calculated the read depth at all the positions, including the RNA editing sites, and found similar trends for all four tissues. We found no difference in the 27 editing sites among the four maize tissues using the Illumina NGS process, and the four editing sites in the *ndhB* gene were the same for the basal stalk and expanded green leaf using Sanger sequencing. Thus, RNA editing does not appear to change among the tissues investigated or depending on light and dark growth conditions.

### Variant Formation and Replication-Transcription Conflict

As shown in Figure 2, the IRs contain replication origins oriA and oriB, terminal sequences End1 and End2, and two prominent variant clusters within the 16S rRNA genic regions. These clusters raise interesting questions. 1) Why are variants clustered in distinct regions of the plastid genome? 2) Why does Cluster 1 (in IRb with 34 variants) contain fewer variants than does Cluster 2 (in IRa with 84 variants), given the exact same nucleotide sequence for IRa and IRb? 3) Why are these two variant clusters only found in the Leaves_dark tissue and not in Leaves_light, L1_light, or Stalk_light tissues?

We propose that the variant clusters arise as a result of replication-transcription conflict and that the larger Cluster two is due to head-on conflict, whereas the smaller Cluster one is a consequence of co-directional conflict. Problems arising from such conflicts have long been studied in bacteria and yeast and if not resolved can lead to genome instability including deletions, rearrangements, and double-strand breaks (DSBs) (Soultanas, 2011; McGlynn et al., 2012; Merrikh et al., 2012). Head-on collision between replisome and the transcription complex is considered more severe than co-directional collision, but the latter can also result in DNA damage. In bacteria, conflict may be minimized by the co-directional orientation of replication from the origin (oriC) and of highly transcribed genes such as the rRNA genes.

**FIGURE 5:**
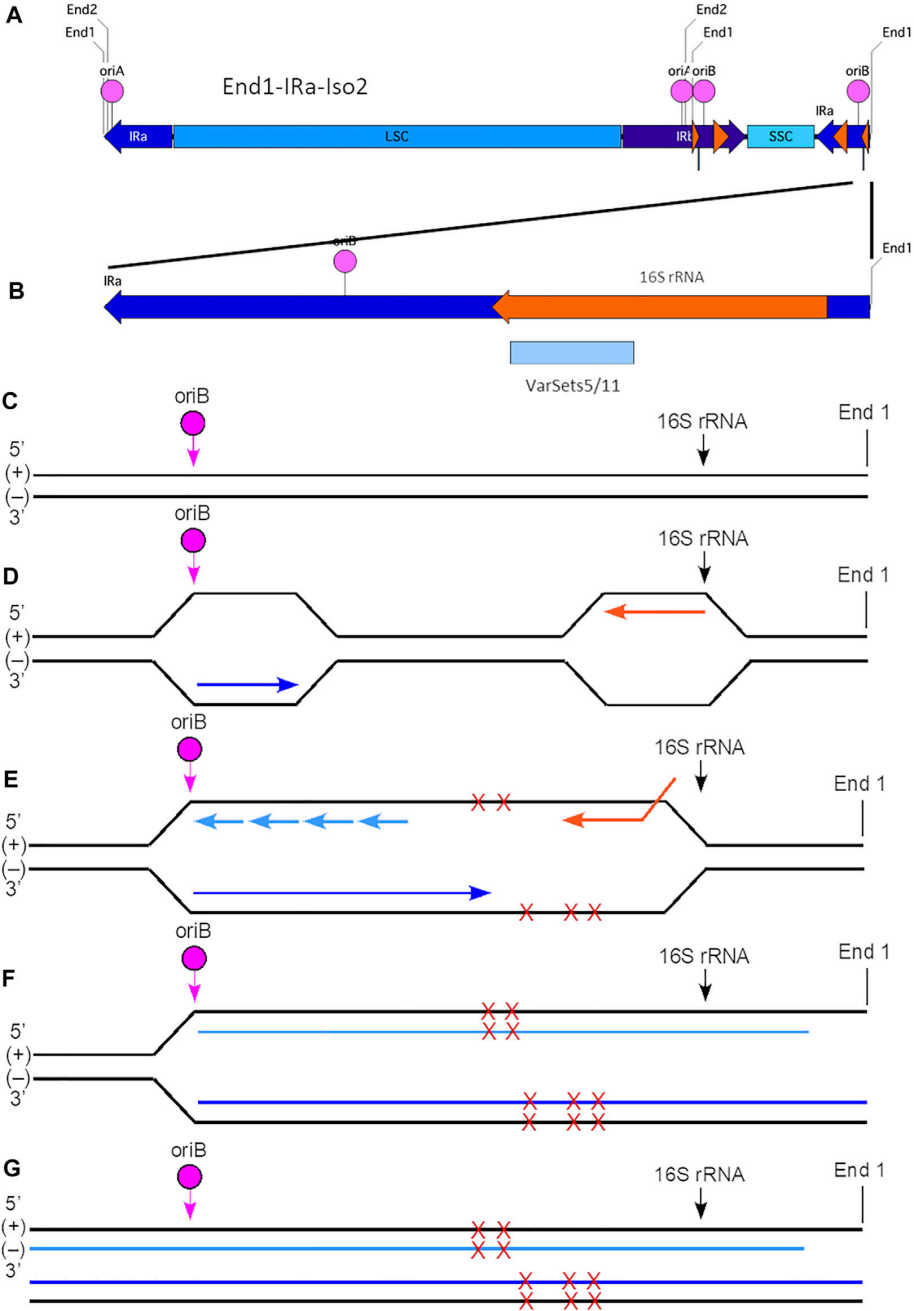
Model for generation of plastid ptDNA variants caused by head-on replication-transcription conflict. **(A)** The plastid DNA molecular isomer used for this model is End1-IRa-Iso2 (see Supplementary Figure S2). In this isoform, the right-end region is comprised of a portion of IRa (nt 117640-127764) that contains oriB (nt 125441-125563), 16S rRNA gene (nt 126089-127579; coding on (−) strand), and with End1 terminus (nt 127764). The variant Cluster 2 (VarSets5/11; nt range 126175-126724) is found within the 16S rRNA genic region of IRa. The nucleotide (nt) numbering corresponds to the maize plastid reference genome. The small orange arrowhead indicates the location of the 16S rRNA gene, and large orange arrowhead the location of the 23S rRNA gene. **(B)** Expanded view of the right-end region of isomer End1-IRa-Iso2, which spans a total of 3,387 nts (nt 124377-127764). The Cluster two region includes 84 variants which span a total of 549 nts and is shown as a blue box below the orange 16S rRNA gene. **(C)** The (+) and (−) strands of the right-end region are shown with the location of oriB and beginning of the 16S rRNA gene indicated. Note that this diagram is not to scale with respect to the distances between oriB, 16S rRNA, and End1 terminus. **(D)** Replication is initiated at oriB in IRa, and the leading-strand replication (indicated by the dark blue arrow) proceeds from left to right and toward the 16S rRNA gene and the right End1 terminus. The 16S rRNA gene coding is on the (−) strand, and transcription (indicated by the orange arrow) proceeds from right to left using the (+) strand template. **(E)** A head-on replication-transcription collision occurs between the advancing replisome and the 16S rRNA transcription machinery that results in extended ssDNA regions that are prone to damage (indicated by red Xs). Multiple sites of damage are shown on both (+) and (−) strands, although it is uncertain whether single or multiple damage points are present for any given molecule. Lagging-strand replication is indicated by short light blue arrows. **(F)** The replication-transcription conflict likely leads to replication stalling and replication fork collapse. With restart, however, replication is resumed and continues toward the right end of the ptDNA molecule. Due to the mechanism of replication, however, a ssDNA 3′-overhang remains at the right end of the (+) strand template. Nonetheless, the damage is incorporated into the nascent ptDNA molecules. **(G)** The end result is complete replication (both strands) of the End1-IRa-Iso2 linear molecule. Depending on the extent of damage that may not be faithfully repaired, variants may arise in one or both of the newly replicated molecules. As shown here, one of these new dsDNA molecules has a ssDNA 3′-overhang. Subsequent recombination-dependent-replication without degradation of the damaged ptDNA molecules in the dark-grown tissue could result in sufficient DNA amplification to be measured as a variant.

Based on work in bacteria, Figure 5 depicts the proposed outcome of head-on collision within the plastid genome. Replication is initiated at oriB in IRa, and the leading-strand replication proceeds from left to right toward the 16S rRNA gene and the right End1 terminus. Transcription of the 16S rRNA gene proceeds from right to left, leading to a head-on collision, replication stalling, and extended ssDNA regions that are susceptible to damage such as point mutations (SNPs). Upon replication restart, the probability of generating deletions/insertions (Indels) increases, adding to the cluster of variants. Replication continues to the right end of the ptDNA molecule. Any uncorrected “damage” (SNP or Indel) is carried over to the newly replicated double-stranded ptDNA molecules. The ssDNA 3′-overhang that remains at End1 of one of the nascent dsDNA molecules can now initiate recombination-dependent-replication (RDR) by single-strand annealing with the internal homologous End1 sequence, in either IRb or IRa, of another ptDNA molecule creating branched molecules observed in DNA moving pictures (Bendich, 2004; Oldenburg and Bendich, 2004a; Oldenburg et al., 2009; Oldenburg and Bendich, 2015). Since the invading molecule contains the unrepaired damage, the SNP/Indel would be copied and incorporated into newly replicated ptDNA. Continued rounds of RDR would generate sufficient molecules containing the same error (damage) to be detectable as a sequence variant. However, most ptDNA would consist of undamaged, wild-type molecules due to replication without conflict-induced damage or by repair of the damage. This inference is supported by our Illumina sequencing data for the dark-grown leaf ptDNA that generate the maize reference genome. In summary, the large variant Cluster two in the IRa region is likely a result of a head-on conflict between replication initiated at oriB and transcription of the 16S rRNA gene.

**FIGURE 6:**
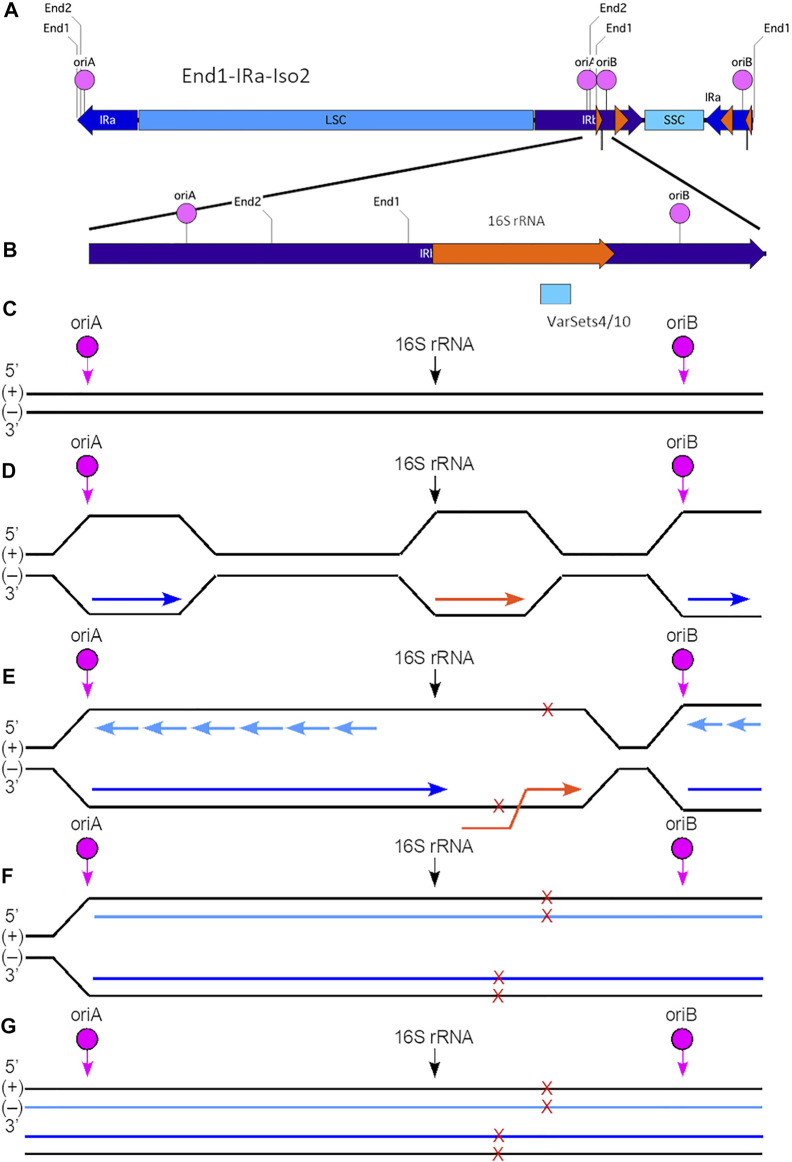
Model for generation of plastid ptDNA variants caused by co-directional replication-transcription conflict. **(A)** The plastid DNA molecular isomer used for this model is End1-IRa-Iso2. In this isoform, the IRb (nt 82356-105103) is located internally between the LSC and SSC and contains both oriA (nt 93170-93455) and oriB (nt 97174-97296). The 16S rRNA gene (nt 95164-96654; coding on (+) strand) is located between these two origins of replication. The variant Cluster 1 (VarSets4/10; nt range 96098-96298) is found within the 16S rRNA genic region. The nucleotide (nt) numbering corresponds to the maize plastid reference genome. The small orange arrowhead indicates the location of the 16S rRNA gene, and large orange arrowhead the location of the 23S rRNA gene. **(B)** Expanded view of a segment of IRb in isomer End1-IRa-Iso2, which spans a total of 5,500 nts (nt 93276-97876). The Cluster one region includes 34 variants which span a total of 200 nts and is shown as a blue box below the orange 16S rRNA gene. **(C)** The (+) and (−) are shown with the location of oriA, oriB and beginning of the 16S rRNA gene indicated. Note that this diagram is not to scale with respect to the distances between oriA, oriB, and 16S rRNA. **(D)** For replication that is initiated at oriA in IRb, the leading-strand replication (indicated by the dark blue arrow) proceeds from left to right and toward the 16S rRNA gene. Replication initiated at oriB also proceeds from left to right, but in this case the leading-strand replication is away from the 16S rRNA gene. The 16S rRNA gene coding is on the (+) strand, and transcription (indicated by the orange arrow) proceeds from left to right using the (−) strand template. **(E)** Replication initiated at oriA may lead to a co-directional replication-transcription conflict within the 16S rRNA gene and result in extended ssDNA regions that are prone to damage (indicated by red Xs). Sites of damage are shown on both (+) and (−) strands, although it is uncertain whether single or multiple damage points are present for any given molecule. Lagging-strand replication is indicated by short light blue arrows. Note that replication generally proceeds faster than does transcription, so that the conflict likely arises as leading-strand replication advances toward the end of the 16S rRNA gene, but before transcription has been completed. **(F)** The replication-transcription conflict likely leads to replication stalling and replication fork collapse. With restart, however, replication is resumed and continues toward the right of the ptDNA molecule. Nonetheless, the damage is incorporated into the nascent ptDNA molecules. **(G)** The end result is complete replication (both strands) of the End1-IRa-Iso2 linear molecule. Depending on the extent of damage that may not be faithfully repaired, variants may arise in one or both of the newly replicated molecules. Subsequent recombination-dependent-replication without degradation of the damaged ptDNA molecules in the dark-grown tissue could result in sufficient DNA amplification to be measured as a variant.

The small variant Cluster one in IRb could result from co-directional replication-transcription conflict, as shown in Figure 6. The 16S rRNA gene in the internal IRb is flanked on the left by oriA and on the on right by oriB. The direction of replication from both oris is from left-to-right as is the direction of transcription of the 16S rRNA gene. Thus, replication initiated at oriB proceeds away from the 16S rRNA gene avoiding conflict. For initiation at oriA, however, the advancing leading-strand replication fork would move toward the transcription complex on the 16S rRNA gene. As shown for bacterial DNA, movement of the replication machinery can be 10–20 times as fast as that of the transcription complex (Brewer, 1988; Merrikh et al., 2012), so that a co-directional conflict could occur if the replication machinery overtakes the transcription complex before transcription of the 16S rRNA gene is completed. And although studies in bacteria and yeast have shown that co-directional conflicts can result in damage, the amount of damage is typically less than with a head-on collision (Soultanas, 2011; Zardoni et al., 2021). The difference in the number of variants within Cluster one in IRb and Cluster two in IRa may therefore be due to the type of replication-transcription conflict (co-directional and head-on, respectively) generated by replication initiated near the 16S rRNA gene. In addition, the chances of conflict and the amount of damage (indicated by the number of variants) could depend upon the transcription activity (high or low) for the 16S rRNA gene and whether a specific ori has been activated in IRa and IRb.

In the bacterium *Bacillus subtilis*, the yeast *Saccharomyces cerevisiae* and *Arabidopsis thaliana* plastids, the generation of excessive (positive) supercoils in DNA, R-loops, and DNA:RNA hybrids develop at the site of a head-on replication-transcription collision (Lang et al., 2017; Yang et al., 2017; Lang and Merrikh, 2021; Zardoni et al., 2021). The type II topoisomerase DNA gyrase in bacteria and plastids and topoisomerase IV in bacteria can mitigate supercoiling and associate preferentially at a site of head-on but not co-directional conflict. In yeast a head-on collision, but not a co-directional collision, leads to an accumulation of R-loops and DNA damage that can be alleviated by the helicase Sen1. In Arabidopsis the plastid-localized RNase H1, AtRNH1C, was shown to maintain plastid genome stability by suppressing R-loops and DNA:RNA hybridization (Yang et al., 2017). Thus, there are as yet uninvestigated agents that may affect variant clustering in maize ptDNA.

We detected the two large variant clusters only for dark-grown seedling leaves, not for any of the other three maize tissues. Why so? During maize development in the light, ptDNA replication and repair are confined to the basal stalk tissue, and during proplastid-to-chloroplast maturation both replication and repair cease, damage increases, and the ptDNA is degraded (Oldenburg and Bendich, 2004b; Oldenburg et al., 2006; Kumar et al., 2014; Tripathi et al., 2020; Tripathi et al., 2022). Thus, we propose that essentially all ptDNA molecules containing sequence variants that might have arisen during replication in Stalk_light would have been either repaired or degraded in the Leaves_light tissue. Any remaining variant molecules would be too infrequent among the “correct” copies to contribute to the sequencing data. In contrast, high genome copy number per plastid and high-integrity molecules persist in etioplasts from dark-grown leaf tissue (Oldenburg et al., 2006; Zheng et al., 2011). We suggest that replication without degradation continues throughout proplastid-to-etioplast development. Accordingly, if replication-transcription conflict leads to damage that is not repaired, continued RDR in etioplasts could result in fixation of this damage detectable as low-copy variants in ptDNA molecules from dark-grown leaves.

## Conclusion

We found the same plastid genome sequence for green leaves that contain highly damaged/fragmented ptDNA molecules and for meristematic cells that contain unfragmented ptDNA molecules. This result is fortunate for applications where total tissue DNA is extracted from green leaves--or herbarium specimens and archeological plant samples--for the study of ptDNA-based phylogenetic relationships. For the study of cell functions that may require interaction among regions widely spaced on a nuclear chromosome, again the molecular integrity of the extracted DNA used for sequencing is unimportant because that chromosome was intact in the nucleus before extraction. For maize, however, it would be incorrect to assume that intact ptDNA molecules contribute to physiological function in the green chloroplast merely because the reference genome is assembled from the shattered ptDNA molecules in chloroplasts.

## Data Availability

The datasets presented in this study can be found in online repositories. The names of the repository/repositories and accession number(s) can be found below: NCBI SRA database (BioProject: PRJNA808651) EVA database (Project: PRJEB51858 Analyses: ERZ6814949).
