# Supplementary material for: Analysis of the Plastid Genome Sequence During Maize Seedling Development
Source: Front Genet. 2022 Apr 26;13:870115. doi: 10.3389/fgene.2022.870115 (PMC9086435; doi:10.3389/fgene.2022.870115)
Supplement: Supplementary file 1 [file DataSheet1.PDF]

Supplementary material 1

Title: Analysis of the plastid genome sequence during maize seedling development

Running title: Plastome genomics in developing maize plants

Authors: Diwaker Tripathi, Delene J. Oldenburg, and Arnold J. Bendich*

Affiliation: Department of Biology, University of Washington, Seattle, WA, United States

***Correspondence:**
Arnold Bendich
(bendich@uw.edu)

Supplementary Table S1. Summary of raw reads alignments against *Zea mays* B73 reference genome using BWA

	Sample ID	Aligned	Paired
1	Leaves_ light #1	67.73%	99.91%
2	Leaves_ light #2	68.03%	99.89%
3	Leaves_dark #1	40.86%	99.95%
4	Leaves_dark #2	40.18%	99.95%
5	Leaf (L1)_light #1	92.09%	99.80%
6	Leaf (L1)_light #2	92.11%	99.80%
7	Stalk_light #1	25.29%	99.97%
8	Stalk_light #2	25.11%	99.97%

Note that Aligned and Paired (Read 1, Read 2) read percentages are calculated based on the total number of reads that passed the filter for each sample.

Supplementary Table S2. Indels/SNPs grouped into Variant sets (VarSet)

The nucleotide position (nt) of the Indel/SNP according to the maize plastid reference genome accession # KF241981.1 is given. REF is the nt sequence from the reference genome, and ALT is the variant sequence as determined by the DRAGEN 3.8 variant caller program. Only variants that passed the DRAGEN QUAL filter were used in the analysis. Indels are highlighted in yellow, and SNPs have no highlighting.

A total of 13 Variant Sets were defined. Variant Set 1 is comprised of three Indels that were common to all four samples. The other Variant Sets were classified according to tissue and biological replicate. There were three "null" sets without any variants - Variant Sets 3, 9, and 12.

For the Leaves_dark tissue, two large variant sets were identified with multiple Indels/SNPs located in "clusters"; Cluster 1 – VarSets4/10 and Cluster 2 –VarSets5/11. The range of nt location for these clusters are given here. See Supplementary Table S3 for specific nt position and base changes. Variant Sets 3 and 9 were initially defined for any variants that would be outliers relative to Clusters 1 and 2. However, these were both null sets.

**Biological Replicate 1**		
**Indels present in all 4 samples**		
Variant set 1		
position (nt)	REF	ALT
3242	GC	G
3754	T	TAA
12961	A	ATTTTTT
**Indels and SNPs in Leaves_light**		
Variant set 2		
position (nt)	REF	ALT
17176	AG	A
**Indels and SNPs in Leaves_dark**		
Variant set 3		
position (nt)	REF	ALT
none		
Variant set 4		
Range		
position (nt)	#Indels	#SNPs
96133 to 96298 IRb	10	20

**Biological Replicate 2**		
**Indels present in all 4 samples**		
Variant set 1		
position (nt)	REF	ALT
3242	GC	G
3754	T	TAA
12961	A	ATTTTTT
**Indels and SNPs in Leaves_light**		
Variant set 8		
position (nt)	REF	ALT
17176	AG	A
**Indels and SNPs in Leaves_dark**		
Variant set 9		
position (nt)	REF	ALT
none		
Variant set 10		
Range		
position (nt)	#Indels	#SNPs
96098 to 96248 Irb	6	12

Variant set 5			Variant set 11		
Range position (nt)	#Indels	#SNPs	Range position (nt)	#Indels	#SNPs
126202 to 126724 IRa	33	46	126175 to 126435 IRa	28	28

**Indels and SNPs in L1_light**			**Indels and SNPs in L1_light**		
Variant set 6			Variant set 12		
position (nt)	REF	ALT	position (nt)	REF	ALT
17176	AG	A	none		

**Indels and SNPs in Stalk_light**			**Indels and SNPs in Stalk_light**		
Variant set 7			Variant set 13		
position (nt)	REF	ALT	position (nt)	REF	ALT
120517	C	CTG	51810	C	CT

Supplementary Table S3. Indels/SNPs found in Leaves_dark tissue

Some variants were unique to Biological replicate 1 or 2. Some variants were common to both replicates; these are indicated by either blue for Cluster 1 – VarSet4/10 or lavender for Cluster 2 – VarSet5/11. Indels are highlighted in yellow, and SNPs have no highlighting.

VarS4/S10	14 Indels/SNP's common to Dark Biological Replicates 1 & 2
VarS4	16 Indels/SNPs unique to Dark Biological Replicate 1
Var S10	4 Indels/SNP's unique to Dark Biological Replicate 2
	For a total of 34 variants that are indicated by dots for Cluster 1 in Figure 2
VarS5/11	51 Indels/SNP's common to Dark Biological Replicates 1 & 2
VarS5	28 Indels/SNP's unique to Dark Biological Replicate 1
VarS11	5 Indels/SNP's unique to Dark Biological Replicate 2
	For a total of 84 variants that are indicated by dots for Cluster 2 in Figure 2

**Leaves_dark Biological Replicate 1**			**Leaves_dark Biological Replicate 2**		
VarSet1			VarSet1		
position (nt)	REF	ALT	position (nt)	REF	ALT
3242	GC	G	3242	GC	G
3754	T	TAA	3754	T	TAA
12961	A	ATTTTTT,ATTTTTTT	12961	A	ATTTTTT
Variant set 3			Variant set 9		
position (nt)	REF	ALT	position (nt)	REF	ALT
none			none		
Variant set 4			Variant set 10		
position (nt)	REF	ALT	position (nt)	REF	ALT
96133	C	A	96098	T	A
96135	C	A	96103	A	C
96138	A	ACTT	96122	G	C
96141	CTCTTGAA	C	96138	A	ACTT
96153	A	AT	96141	CTCTTGAA	C
96155	G	GATT	96148	A	ACTTTCC
96162	C	T	96153	A	AT
96171	G	T	96155	G	GATT
96173	G	T	96162	C	T
96184	G	C	96171	G	T
96208	C	T	96173	G	T
96212	A	G	96184	G	C
96215	G	A	96208	C	T
96230	T	C	96212	A	G

96233	C	T
96248	TCG	T
96253	T	TCC
96260	G	A
96263	A	AG
96264	C	CACGT
96268	GA	G
96270	GT	G
96273	T	G
96276	A	C
96279	C	T
96282	G	A
96284	A	G
96285	C	G
96290	C	T
96298	T	A

96215	G	A
96230	T	C
96233	C	T
96248	TCG	T

Variant set 5		
position (nt)	REF	ALT
126202	T	C
126208	C	CAT
126209	GC	G
126213	G	T
126215	C	A
126216	T	C
126217	AG	A
126221	T	G
126222	TA	T
126226	C	T
126228	T	A
126230	C	CG
126231	C	T
126233	CTCCTTA	C
126236	CTTA	C
126241	GGTT	G
126249	G	T
126251	AAT	A
126257	TTCA	T
126261	A	AGCT
126263	C	CTT
126267	G	GTGCAAC
126270	AG	A
126276	T	C
126279	A	G
126314	G	T
126323	C	CGA

Variant set 11		
position (nt)	REF	ALT
126175	A	C
126176	G	C
126202	T	C
126208	C	CAT
126209	GC	G,GAATCAC
126211	AAG	A
126215	C	CGTGG,A
126220	CTTA	C
126222	TA	T
126224	GGCA	G
126226	C	T
126228	T	A
126230	C	CG
126231	C	T
126233	CTCCTTA	C
126236	CTTA	C
126241	GGTT	G
126249	G	T
126251	AAT	A
126257	TTCA	T
126261	A	AGCT
126263	C	CTT
126267	G	GTGCAAC
126270	AG	A
126276	T	C
126279	A	G
126314	G	T

126324	CGT	C
126328	TG	T
126330	G	T
126334	A	ATT
126335	CCG	C
126356	T	G
126357	G	A
126363	T	C
126368	G	T
126379	C	A
126384	A	G
126390	A	G
126393	TGAGG	TAGG,T
126396	G	C
126398	A	AT
126400	G	GATC
126401	GGT	G
126406	T	TA
126408	G	GT
126410	A	G
126411	G	GA,A
126419	A	C
126420	C	A
126428	A	G
126429	G	C
126430	A	T
126432	C	G
126435	G	A
126593	A	G
126608	C	T
126611	T	C
126615	G	A
126639	C	G
126646	T	TATGAAA
126648	T	TAAGTA
126650	CGCG	C
126654	T	A
126659	GAGGGCACCCCTCTCT	G
126677	C	A
126680	G	GAC
126684	A	T
126687	C	T
126689	C	T
126690	G	T
126691	G	GTT

126323	C	CGA
126324	CGT	C
126328	TG	T
126330	G	T
126334	A	ATT
126335	CCG	C
126356	T	G
126357	G	A
126363	T	C
126368	G	T
126379	C	A
126384	A	G
126390	A	G
126393	TGAGG	TAGG,T
126396	G	C
126398	A	AT
126400	G	GATC
126401	GGT	G
126406	T	TA
126408	G	GT
126410	A	G
126411	G	GA,A
126419	A	C
126420	C	A
126428	A	G
126429	G	C
126430	A	T
126432	C	G
126435	G	A

126692	C	CAT
126701	C	G
126702	C	G
126714	C	T
126716	T	G
126720	T	G
126724	C	T

Supplementary Figure S1

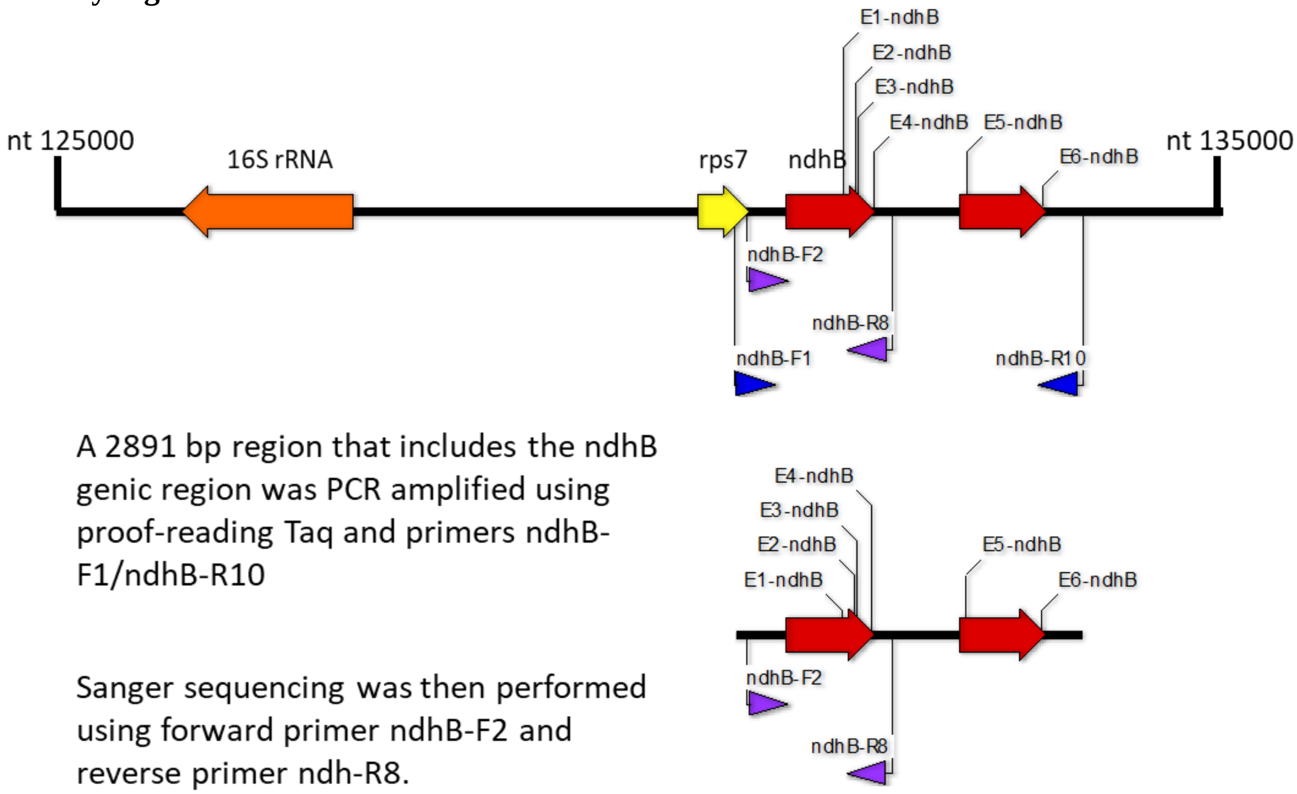

Figure S1. Diagrams of a section of the maize plastid genome including the *ndhB* genic region. A section of the maize plastid genome that spans nts 125000-135000 of the IRa region is depicted with the 16S rRNA (orange), *rps7* (yellow), and exon 1 and 2 of *ndhB* (red) genes shown. In addition, the six RNA editing sites within the *ndhB* gene and PCR primers (purple and blue arrowheads) are indicated. Primers ndhB-F1 (5'-AAGCATCCCAAAAGCGTCC-3') and ndhB-R10 (5'-CAAAAGCAGGTCTGATTACACC-3') were used with high-fidelity Taq DNA polymerase to amplify a region spanning the *ndhB* gene with ptDNA isolated from L1_light and Stalk_light tissues. Sanger sequencing was then performed using these 2891-bp PCR fragments with the forward primer ndhB-F2 (5’-CTCATAGAATGGCAGAGGC-3’) and the reverse primer ndhB-R8 (5’-GGTAAAAGTTCTGTCTTGGTCG-3’). The numbering of the nt positions corresponds to the B73 reference genome (accession KF241981.1).

Supplementary Figure S2

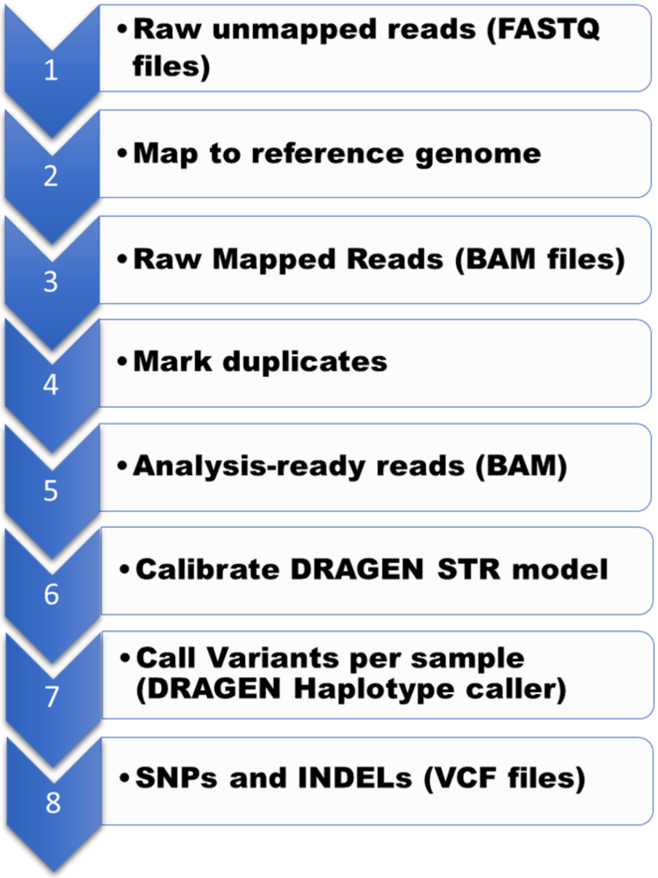

Figure S2. Flowchart of DRAGEN germline variant analysis

Supplementary Figure S3

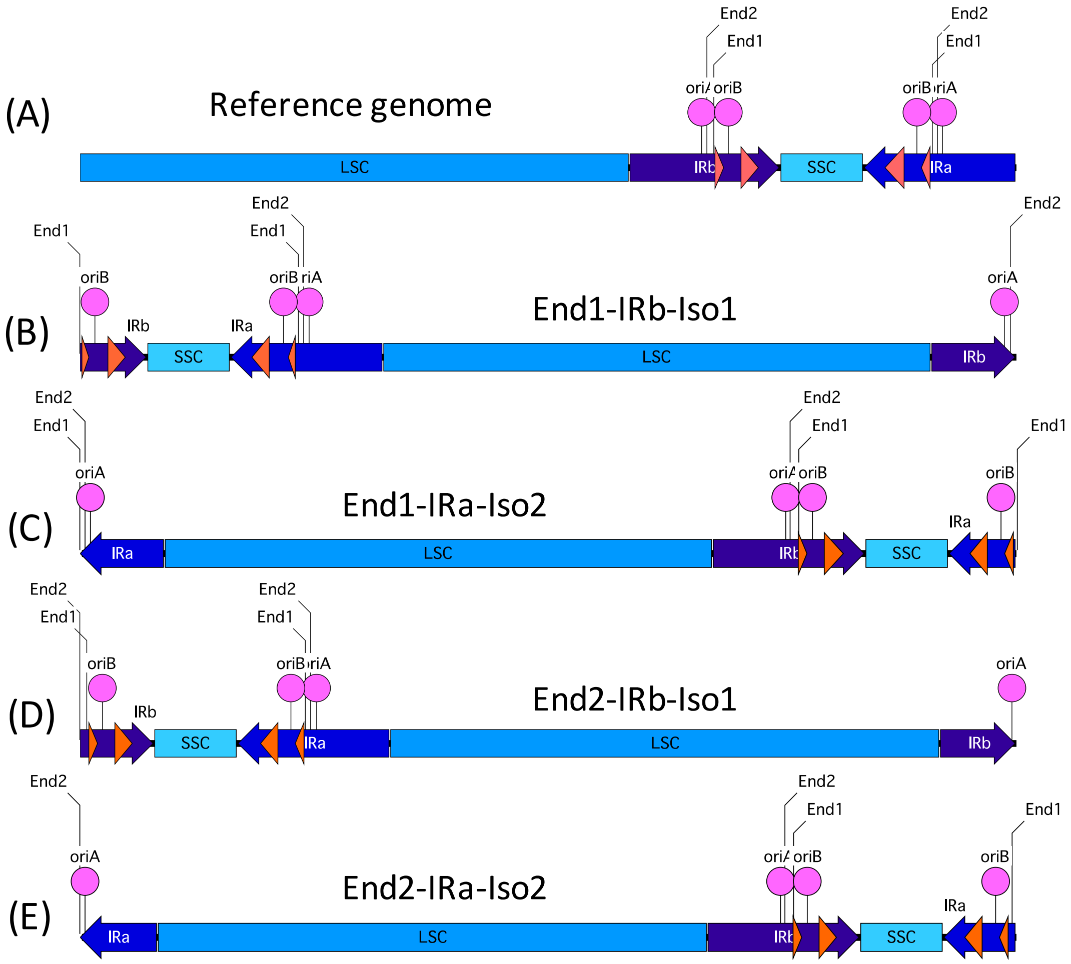

Figure S3. Diagrams of the maize plastid genomic isomers
Like most plants the maize plastid genome has the arrangement of long- and short-single copy regions (LSC and SSC, respectively), separated by a pair of inverted repeats (IRs, IRb and IRa). (A) The arrangement of the B73 plastid reference genome is depicted using the convention of beginning in the LSC region and ending in the IRa region. These are not, however, the actual ends/termini of the maize ptDNA linear molecules. Two discrete end sequences (End1 and End2), both located in the IRs, were identified by cloning and sequences (Oldenburg and Bendich, 2016 Curr Genet 62:431-442). Thus, maize ptDNA is comprised of a collection molecular isomers with specific ends and four of these isomers are shown here. There are two isomers with the End1 sequence, (B) End1-IRb-Iso1 and (C) End1-IRa-Iso2, and two with the End2 sequence, (D) End2-IRb-Iso1 and (E) End2-IRa-Iso2. For each diagram the position of the ends are indicated by lines, the origins of replication, oriA and oriB, by pink dots, and the 23S and 16S rRNA genes by large and small orange arrowheads, respectively.

Supplementary Figure S4

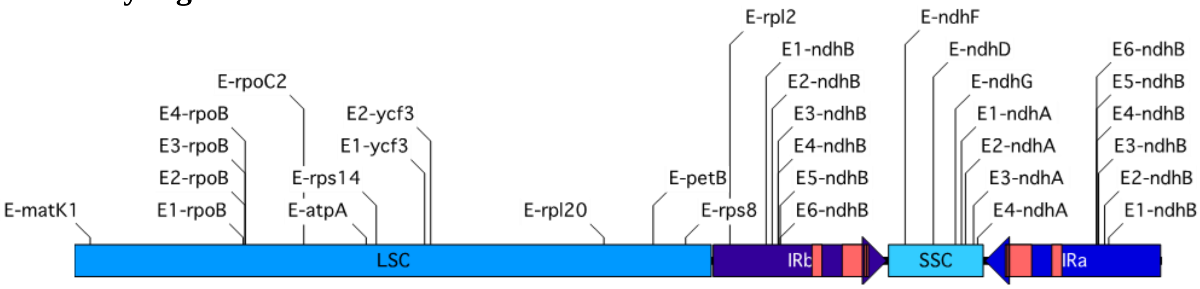

Editing sites	Position
matK-1	1954
rpoB	21924
rpoB	22002
rpoB	22017
rpoB	22074
rpoC2	29748
atpA	37711
rps14	38885
ycf3	45258
ycf3	46135
rpl20	68327
peTB	74514
rps8	78726
rpl2	84436
ndhB	89313
ndhB	89964
ndhB	90761
ndhB	90887
ndhB	90912
ndhB	91031
ndhF	107268
ndhD	110808
ndhG	113578
ndhA	114407
ndhA	114914
ndhA	116027
ndhA	116450

Position of RNA editing sites for ndhB gene

	in IRb	in IRa
E1	89313	133452
E2	89964	132801
E3	90761	132006
E4	90887	131878
E5	90912	131853
E6	91031	131734

Figure S4. Diagram and list of RNA editing sites in the maize plastid genome.
A diagram of the maize B73 plastid reference genome is shown at the top and corresponds to the standard arrangement with the first nucleotide (nt1) at the beginning of the LSC. The positions of the 27 RNA editing are indicated on the diagram. A list of the RNA edit site within a specific gene and position (nt) is shown. Since the *ndhB* gene is located in the IRs, the position in both IRb and IRa for these six sites is also provided. The numbering of the nt positions corresponds to the B73 reference genome (accession KF241981.1) (Bosacchi et al., 2015, Plant Physiology 169 (3), 2129-2137.
